# The Hierarchical Sequence Requirements of the H1 Subtype-Specific Noncoding Regions of Influenza A Virus

**DOI:** 10.1128/spectrum.03153-22

**Published:** 2022-10-26

**Authors:** Minglei Pan, Wenyu Zhang, Yue Xiao, Yuerong Lai, Mengmeng Cao, Jianwei Wang, Tao Deng

**Affiliations:** a NHC Key Laboratory of Systems Biology of Pathogens, Institute of Pathogen Biology, Chinese Academy of Medical Sciences and Peking Union Medical College, Beijing, China; b CAS Key Laboratory of Pathogen Microbiology and Immunology, Institute of Microbiology, Chinese Academy of Sciences, Beijing, China; University of Nevada, Reno

**Keywords:** influenza A virus, segment-specific and subtype-specific noncoding nucleotides, virus random nucleotide selection assay, sequence requirements, promoter-proximal nucleotides

## Abstract

The genome of influenza A virus consists of eight single-stranded viral RNA (vRNA) segments. The nonconserved noncoding regions (NCRs) at the 3′ and 5′ termini of each segment show extremely low divergence and mutation rate. They appear as segment specific among the eight segments and also subtype specific among different subtype-determinant hemagglutinin (HA) and neuraminidase (NA) segments. In order to acquire in-depth knowledge on the sequence requirements of the segment-specific or subtype-specific NCRs (ssNCRs), we, in the context of WSN (H1N1) reverse genetics, designed a virus random nucleotide selection assay (vRNSA) in which we generated pHW2000-HA plasmid libraries with random nucleotides in each grouped nucleotide positions in the 3′ and 5′ H1-ssNCRs, followed by virus rescue, serial passage, and deep sequencing. The resulting sequence logos present a visualized dynamic overview of the hierarchical sequence requirements of the 3′ and 5′ H1-ssNCRs. It showed that, in the process of continuous passage, the 3′ H1-ssNCR, in general, stabilized more quickly than the 5′ H1-ssNCR. The nucleotides close to the highly conserved 3′ and 5′ promoter regions showed higher sequence stringency than nucleotides away from the promoter regions. All stabilized sequences displayed a common feature of high A/U ratios. Especially with our mutational function analyses, we demonstrate that the 3′ promoter-proximal nucleotides could cooperatively exert a direct effect on the transcription and replication of the HA segment. Together, these results provide in-depth knowledge for understanding the NCRs of influenza A virus.

**IMPORTANCE** The segment-specific and subtype-specific nonconserved noncoding regions (ssNCRs) at both 3′ and 5′ ends of viral RNA segments of influenza A virus are largely conserved among the same segments of different viruses. However, the function-related sequence requirements of these ssNCRs remain unclear. In this study, through a novel self-designed vRNSA approach, we present a visualized dynamic overview diagram directly reflecting the hierarchical sequence requirements within and between the 3′ and 5′ H1-ssNCRs. The in-depth functional mutagenesis analyses further revealed that specific nucleotides in the 3′ promoter-proximal region could cooperatively exert a direct effect on viral RNA transcription and replication. This work further advanced our knowledge in understanding the nonconserved noncoding regions of influenza A viruses.

## INTRODUCTION

Influenza A viruses are highly infectious respiratory pathogens posing a great threat to public health (1). Influenza A virus is an eight-segmented, negative-sense RNA virus of the family *Orthomyxoviridae*. Within the virions, each viral RNA segment is present in the form of viral ribonucleoprotein complexes (vRNPs) in which the RNA associates with nucleoprotein (NP) oligomers and a heterotrimeric RNA-dependent RNA polymerase formed by polymerase basic 1 (PB1), PB2, and polymerase acid (PA) (2). The vRNPs are the minimal functional unit required for viral RNA transcription (vRNA→mRNA) and replication (vRNA↔cRNA) to occur in the nucleus of infected cells (3). The strategies used by the viral RNA polymerase to initiate and terminate the transcription and replication are very different (4). The initiation of vRNA→mRNA synthesis is primer dependent involving a host cap-snatching process while the initiations of vRNA↔cRNA syntheses are primer independent but with internal initiation on the cRNA promoter and terminal initiation on the vRNA promoter (5–7). The termination of mRNA synthesis occurs by repeatedly copying a stretch of U close to the 5′ end of vRNA leading to viral mRNA polyadenylation (8), while the replication is the full-length copy between vRNA and cRNA. Both vRNAs and cRNAs are assembled into vRNPs and cRNPs along with their syntheses (9). When sufficient vRNPs are produced in the nucleus, they get exported to the cytoplasm and travel across cytoplasm to the plasma membrane where they are selectively packaged into progeny virions (10, 11).

The eight vRNA segments of the influenza genome, designated polymerase basic 2 (PB2), polymerase basic 1 (PB1), polymerase acid (PA), hemagglutinin (HA), nucleoprotein (NP), neuraminidase (NA), matrix (M), and nonstructural protein (NS), contain one or two major open reading frames (ORFs) in negative sense, which are flanked by noncoding regions (NCRs) at both ends. The NCRs of each segment consist of a highly conserved promoter region (12 and 13 nucleotides from the 3′ and 5′ ends, respectively) followed by the nonconserved noncoding region that are segment specific among the eight segments and subtype-specific NCRs among the two subtype-determinant HA and NA segments (12, 13). These segment-specific or subtype-specific NCRs (ssNCRs) vary significantly in sequence and length (from 5 to 45 nucleotides), at the 3′ and 5′ ends of different segments (13–16). It has been reported that, compared with the coding region, these ssNCRs are largely conserved in the same vRNA segments of different influenza virus strains with extremely low divergence and evolutionary rate (17).

It has been demonstrated that segment-specific NCRs play multiple roles in the life cycle of influenza A viruses. The 3′- and 5′-segment-specific NCRs together with adjacent terminal coding regions have been reported for each segment acting as selective packaging signals (18–27). The replacement of H1-subtype-specific NCRs with other subtype-specific NCRs at both ends or at single ends in the context of H1N1 virus have suggested that the 3′ HA ssNCR plays a more critical role than the 5′ HA ssNCR in regulating virus multiplication, and no stringent compatibility between the two ends is required (13). In addition, earlier studies have shown that the simultaneous deletions of ssNCRs at both ends in the NA segment of the influenza virus A/WSN/33 (H1N1) significantly reduced the replication of this segment (28). Moreover, the HA-specific nucleotides in the 3′ and 5′ ssNCRs forming the extended duplex region of the promoters were found to play dual roles in regulating RNA syntheses and controlling virus selective genome packaging (16). Recently, we have reported that the terminal noncoding and adjacent coding regions act synergistically to ensure optimal levels of HA vRNA replication in a multisegment environment (29). However, the sequence requirements of these ssNCRs remain unclear.

In this study, we aimed to characterize the sequence requirements of the ssNCRs in the context of the H1 segment of influenza A virus. We designed a novel virus random nucleotide selection assay (vRNSA) to explore the nucleotide selectivity of the H1-ssNCRs during virus passage. Through this assay and our in-depth functional studies, we have obtained in-depth knowledge in understanding the basic characteristics of H1-ssNCRs. An A19U mutant and the appearance of its recovery U23G mutation further highlight the essentiality of the 3′ promoter-proximal nucleotides in regulating viral RNA transcription and replication.

## RESULTS

### The hierarchical sequence requirements within and between the 3′ and 5′ H1-ssNCRs upon virus serial passage.

**FIG 1:**
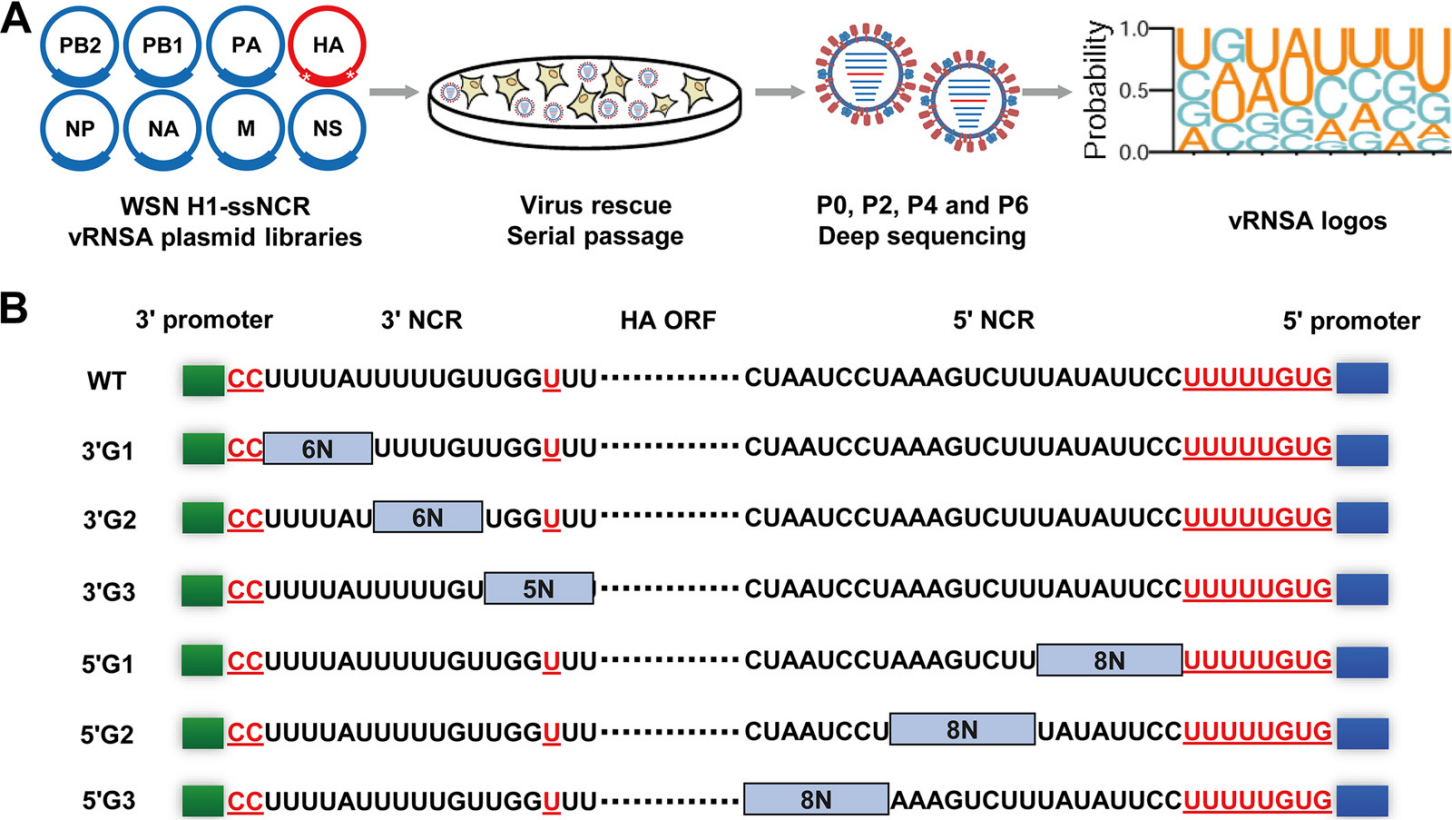
The virus random nucleotide selection assay (vRNSA) identifies hierarchical sequence requirements of the 3′ and 5′ H1-ssNCRs of the WSN virus. (A) The flow chart of the vRNSA. The white star represents the random mutation region. (B) Schematic representation of random mutations at the 3′ and 5′ H1-ssNCRs of the WSN HA segment. The green and blue boxes represent the 12 and 13 terminal promoter nucleotides at the 3′ and 5′ ends of HA vRNA, respectively. The dotted line represents HA ORF. The nucleotides shown between the promoter and ORF are the subtype-specific NCRs of the HA segment of the WSN virus. The bases marked red and underlined are found to be important for virus replication in previous studies and were unchanged in this study. The gray squares represent mutation regions of the vRNSA.

To explore the sequence requirements of H1-ssNCRs for virus propagation, in the context of WSN (H1N1) reverse genetics, we designed a virus random nucleotide selection assay (vRNSA) in which we provided random nucleotides at each nucleotide position in the 3′ and 5′ H1-ssNCRs, followed by virus rescue passage 0 (P0); six generation passages in Madin-Darby canine kidney (MDCK) cells; and deep sequencing at P0, P2, P4, and P6 (Fig. 1A). Considering the limitations of transformation efficiency in plasmid preparation and transfection efficiency during virus rescue, we divided the 3′ and 5′ H1-ssNCR into three groups. Each 3′ H1-ssNCR group contained six random nucleotides (3'G1 [15 to 20], 3'G2 [21 to 26], and 3'G3 [27 to 32]), and each 5′ H1-ssNCR group contained eight random nucleotides [5'G1] [22' to 29'; termed 22' to 29' to distinguish them from 3′ group], (5'G2 [30' to 37'], and 5'G3 [38' to 45']) (Fig. 1B). For those nucleotides with essential functions were kept unchanged, such as a U nucleotide at −3 position before the start codon, the HA-specific CC nucleotides adjacent to the 3′ promoter region, and the stretch of U, the HA-specific GUG nucleotides adjacent to the 5′ promoter region (12, 16, 30). Based on this principle, we generated six pHW2000-HA rescue plasmid libraries (3'G1, 3'G2, 3'G3, 5'G1, 5'G2, and 5'G3) with random nucleotides in each group by using primers containing degenerate bases in each nucleotide position.

**FIG 2:**
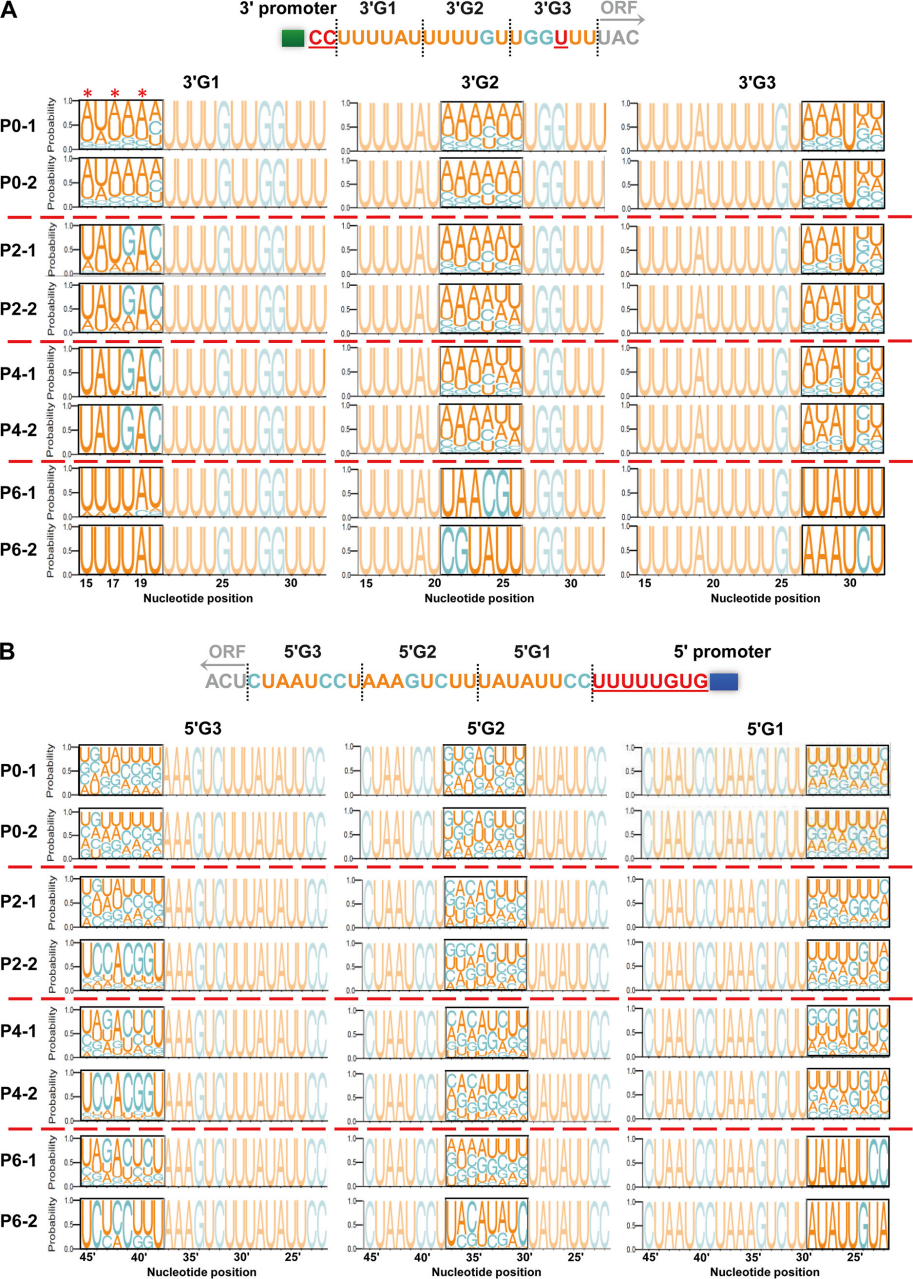
The sequence logos of the WSN H1-ssNCRs generated by the vRNSA. (A and B) Random nucleotide sequences are introduced at indicated regions (3'G1, 3'G2, 3'G3, 5'G1, 5'G2, and 5'G3) from Fig. 1B and the recombinant viruses were rescued. The recombinant virus was passaged for six generations on MDCK cells, and the P0, P2, P4, and P6 generations viral particle RNA genomes were sequenced. The mutation regions of the vRNSA are marked by black boxes. The height of the letter in the logos is in proportion to the frequency of the nucleotide at that position. The red asterisks mark the nucleotides at positions 15, 17, and 19 that are more rapidly stabilized to the wild-type sequence. The data show two independent experiments.

We then conducted the vRNSA with these plasmid libraries in two independent experiments. Considering that the untranslated regions of influenza viruses have an extremely low divergence and evolutionary speed (17), together with the fact that they are very stable during passages in cell culture, we did not perform next-generation sequencing (NGS) for the wild-type (WT) WSN virus. The NGS results of the rescued viruses with randomly mutated NCRs showed that, in the first round of the virus rescue (P0), the nucleotides in 5'G3 and 5'G2 at P0 are completely in a disordered state with the relatively equal ratio of the four types of nucleotides (Fig. 2B), confirming that the nucleotide randomization has worked well and the mutagenesis was not biased. The relatively unequal ratios of the four nucleotides shown in 3'G1, 3'G2, 3'G3, and 5'G1 indicate that the selection has obviously occurred during the initial virus rescue step (Fig. 2A and B). Fig. 2A showed that, within the 3′ H1-ssNCR, all nucleotides in the three 3'G1, 3'G2, and 3'G3 groups were stabilized into a constant sequence at P6. However, the stabilization speed of the nucleotide sequence in 3'G1 was much faster than that in 3'G2 and 3'G3. Moreover, the stabilized sequence in 3'G1 was exactly the same as that in the wild-type virus in both replicates, whereas the stabilized sequences in 3'G2 and 3'G3 not only differed from the wild-type sequence but also differed in the two replicates. Fig. 2B showed that, within the 5′ H1-ssNCR, only the nucleotides in 5'G1 were stabilized to a constant sequence at P6 in which the two replicates showed one wild-type sequence and one non-wild-type sequence. For those nucleotides distant from the 5′ promoter in 5'G2 and 5'G3, they were still in chaotic sequence. These results suggest strongly that the nucleotides close to the 3′ and 5′ promoters require higher sequence stringency than those nucleotides distant from the 3′ and 5′ promoter. On the other hand, the extents of sequence flexibility were significantly different between the 3′ and 5′ H1-ssNCRs (compare Fig. 2A and B) in which the 5′ H1-ssNCR showed much higher sequence variation than the 3′ H1-ssNCR. This result unveils the hierarchical sequence requirements within and between the 3′ and 5′ H1-ssNCRs.

### The stabilized sequences are sufficient for virus replication in cell culture and show a feature of high A/U ratios.

**FIG 3:**
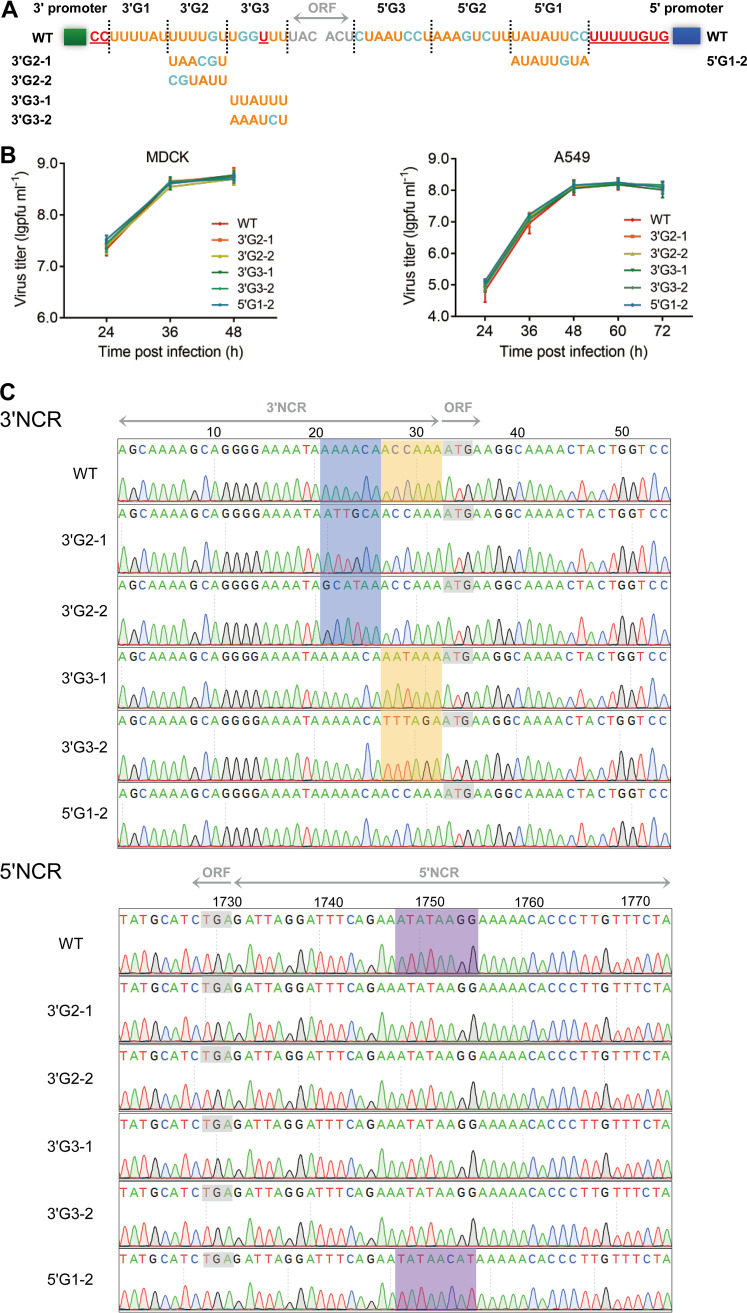
Effects of the stabilized sequences of the H1-ssNCRs on WSN virus replication. (A) Schematic representation of the stabilized sequence. The green and blue boxes represent the 12 and 13 terminal promoter nucleotides at the 3′ and 5′ ends of HA vRNA, respectively. The two-way arrow represents the HA ORF. (B) Replication kinetics of wild-type and mutant viruses in MDCK and A549 cells. MDCK or A549 cells were infected with wild-type or mutant viruses at an MOI of 0.001, and the supernatants were harvested at indicated time points. The virus titers were determined by plaque assay. Data represent the mean ± SD; *n* = 3 biological replicates. (C) Wild-type and mutant viruses were passaged for six generations on MDCK cells, and viral particle RNA genomes were sequenced. Reverse cDNA sequence traces of the 3′ and 5′ terminus HA vRNA of the wild-type and mutant viruses. Start codons and termination codons are marked with gray boxes. The 3'G2, 3'G3, and 5'G1 regions are marked with blue, yellow, and purple boxes, respectively.

Since the stabilized sequences in 3'G2, 3'G3, and 5'G1 showed different sequences from the wild type, we then went on to examine the effects of these non-wild-type sequences on virus replication efficiencies and their stability along serial passage in cell culture. With the reverse genetics, we generated five mutant viruses containing the stabilized non-wild-type sequences as shown in Fig. 3A. The simultaneously rescued wild-type and mutant WSN viruses were then passaged for two generations in MDCK cells, and the replication kinetics of wild-type and mutant viruses was examined in MDCK or A549 cells. As expected, the growth curves of all mutant WSN viruses in MDCK and A549 cells were similar to those of the wild-type virus (Fig. 3B). We subsequently plaque purified the mutant viruses, passaged them for six generations, and sequenced both the 3′ and 5′ NCRs of the HA segment. The results showed that the mutant sequences remained stable and no other mutations were generated in the 3′ and 5′ NCRs of the HA segment (Fig. 3C). These results illustrate that the stabilized non-wild-type sequences selected by the virus are sufficient for efficient virus replication in the cell culture.

Next, we analyzed the nucleotide composition of these stabilized non-wild-type sequences. The results showed that the proportion of A and U bases to the total bases (A/U ratio) was above 4/6 in the stabilized mutant sequences in the 3′ and 5′ H1-ssNCRs, e.g., the 3'G3-1 group was entirely composed of A and U bases (Table 1). In addition, the A/U ratios of the corresponding wild-type sequences were all higher than 4/6. Overall, the results above suggest that the H1-ssNCR nucleotides display a higher requirement of the proportion of A and U bases than the actual sequences.

**TABLE 1:** The ratio of A and U nucleotides of stabilized sequences in the H1-ssNCRs

Virus	Sequence (A/U ratio^*a*^) by group				
	3'G2-1	3'G2-2	3'G3-1	3'G3-2	5'G1-2
Passage 6 mutant	UAACGU (4/6)	CGUAUU (4/6)	UUAUUU (6/6)	AAAUCU (5/6)	AUAUUGUA (7/8)
WT	UUUUGU (5/6)		UGGUUU (4/6)		UAUAUUCC (6/8)

a A/U ratio represents the proportion of A and U bases to the total bases.

### A point mutation introduced at a fast-stabilized position A19U affects viral replication efficiency.

Since the wild-type sequence was selected with an extremely high A/U ratio in 3'G1 at P6 in the two independent replicates, together with the observation that 15U, 17U, and 19A in 3'G1 were the fastest stabilized in P2 and remained stable from P2 to P6 passages, we were interested in examining the effects of these As or Us in regulating virus replication. To avoid changing the A/U ratio mentioned earlier, we mutated nucleotides 15U, 17U, and 19A into 15A, 17A, and 19U, respectively.

**FIG 4:**
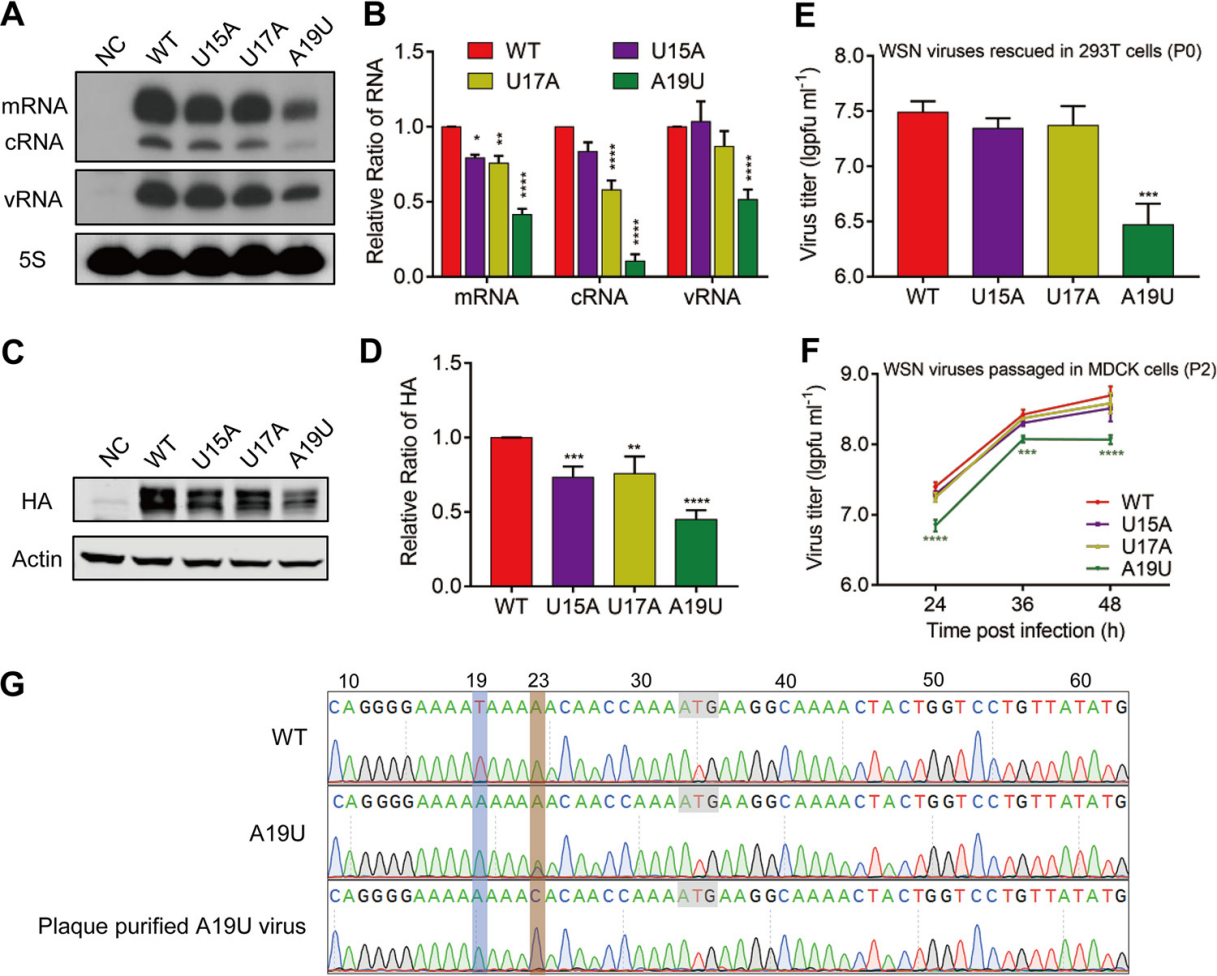
Nucleotide 19A in the 3′ H1-ssNCR is essential for viral replication efficiency. (A) Effect of nucleotide substitutions at positions 15, 17, and 19 in the HA ssNCR 3'G1 group on viral RNA synthesis. 293T cells were transfected with four protein expression plasmids (pcDNA-PA, -PB1, -PB2, and -NP), together with a wild-type or mutant viral RNA expression plasmid (pPOLI-HA). A mutant polymerase containing active site mutations (PB1a: D445A/446A) was used as a negative control (NC). The levels of mRNA, cRNA, and vRNA were detected by primer extension at 24 h posttransfection. (B) Statistical analysis of the viral RNAs in. The values of viral RNAs were standardized to the 5S rRNA level and then normalized to the levels of viral RNAs in the wild type. (C) Effect of nucleotide substitutions at positions 15, 17, and 19 in the HA ssNCR 3'G1 group on viral protein expression. 293T cells were transfected as indicated for A. The levels of HA protein were analyzed by Western blotting at 24 h posttransfection. β-Actin was detected as a loading control. (D) Quantification of the HA protein levels obtained from the experiments shown in C by densitometry analysis. (E) Titers of wild-type and mutant WSN rescued viruses (P0). Wild-type or mutant viruses were rescued in HEK-293T cells by cotransfecting eight pHW2000-PB2, -PB1, -PA, -HA (wild type or mutant), -NP, -NA, -M, and -NS plasmids. The supernatants were harvested at 48 h posttransfection, and the titers were determined by plaque assay. The graph shows the mean titers of wild-type and mutant viruses. (F) Replication kinetics of wild-type and mutant viruses in MDCK cells. MDCK cells were infected with P2 generation wild-type or mutant viruses at an MOI of 0.001, and the supernatants were harvested at indicated time points. The virus titers were determined by plaque assay. (G) Reverse cDNA sequence traces of the wild type, A19U mutant viruses, and plaque-purified A19U virus from F. Start codons are marked with gray boxes. Nucleotides at position 19 and 23 are shown in blue and brown boxes, respectively. All data represent the mean ± SD; *n* = 3 biological replicates. *, *P* < 0.05; **, *P* < 0.01; ***, *P* < 0.001; ****, *P* < 0.0001.

First, we examined the effects of these mutants on viral RNA synthesis and protein production in an RNP reconstitution system derived from the WSN virus (31). The wild-type or mutant pPOLI-HA RNA-expressing plasmid was individually cotransfected with pcDNA-PB2, pcDNA-PB1, pcDNA-PA, and pcDNA-NP plasmids in 293T cells. At 24 h posttransfection, the total RNA of the cells was extracted, and the levels of three viral RNA species (mRNA, cRNA, and vRNA) were detected by primer extension analysis. As shown in Fig. 4A and B, U15A and U17A mutations caused a slight reduction in mRNA and cRNA levels and had no effect on vRNA level, whereas the A19U mutation significantly attenuated the level of all three species of viral RNAs; in particular, cRNA showed 10% of the wild-type cRNA level. We also performed Western blotting to examine the protein production with a monoclonal anti-HA antibody. Consistent with the mRNA levels, U15A and U17A mutations caused a slight decrease in the levels of HA protein, while the A19U mutation caused significantly reduced HA protein levels (>50%) (Fig. 4C and D).

Next, we examined the effect of these mutations on viral replication efficiency. The wild-type and mutant viruses were rescued with the WSN reverse genetics, and the titers of the rescued viruses (P0) were examined by plaque assay. The U15A and U17A mutant viruses showed similar titers to the wild-type virus, while the viral titer of A19U decreased approximately 10-fold compared with that of the wild type (Fig. 4E). To further characterize these mutant viruses, the rescued wild-type or mutant WSN virus (P0) was then passaged for two generations in MDCK cells, and the growth kinetics of the wild-type and mutant viruses (P2) were determined in MDCK cells. The results showed that the growth kinetics of U15A and U17A viruses were similar to that of the wild-type virus; interestingly, the titer of A19U virus decreased by only 4-fold compared with the wild-type virus (Fig. 4F). To address the molecular basis of the improved growth, we sequenced the HA vRNA of the A19U virus (P2). In the A19U virus, point mutation U23G (A23C in positive sense) was found (Fig. 4G). Subsequently, we plaque purified the P2 A19U mutant viruses and sequenced the HA segment individually. We found that the U23G mutation exists simultaneously with the A19U mutation, and no individual U23G mutant virus was found. Taken together, these results suggest that the 3′ 19A nucleotides in H1-ssNCR are under the highest selection pressure during virus propagation. In addition, the A19U mutant virus is unstable and can induce a mutation at its adjacent position upon passaging, which could improve its growth.

### A reverted U23G mutation neutralizes the effects of the A19U mutation during virus replication.

**FIG 5:**
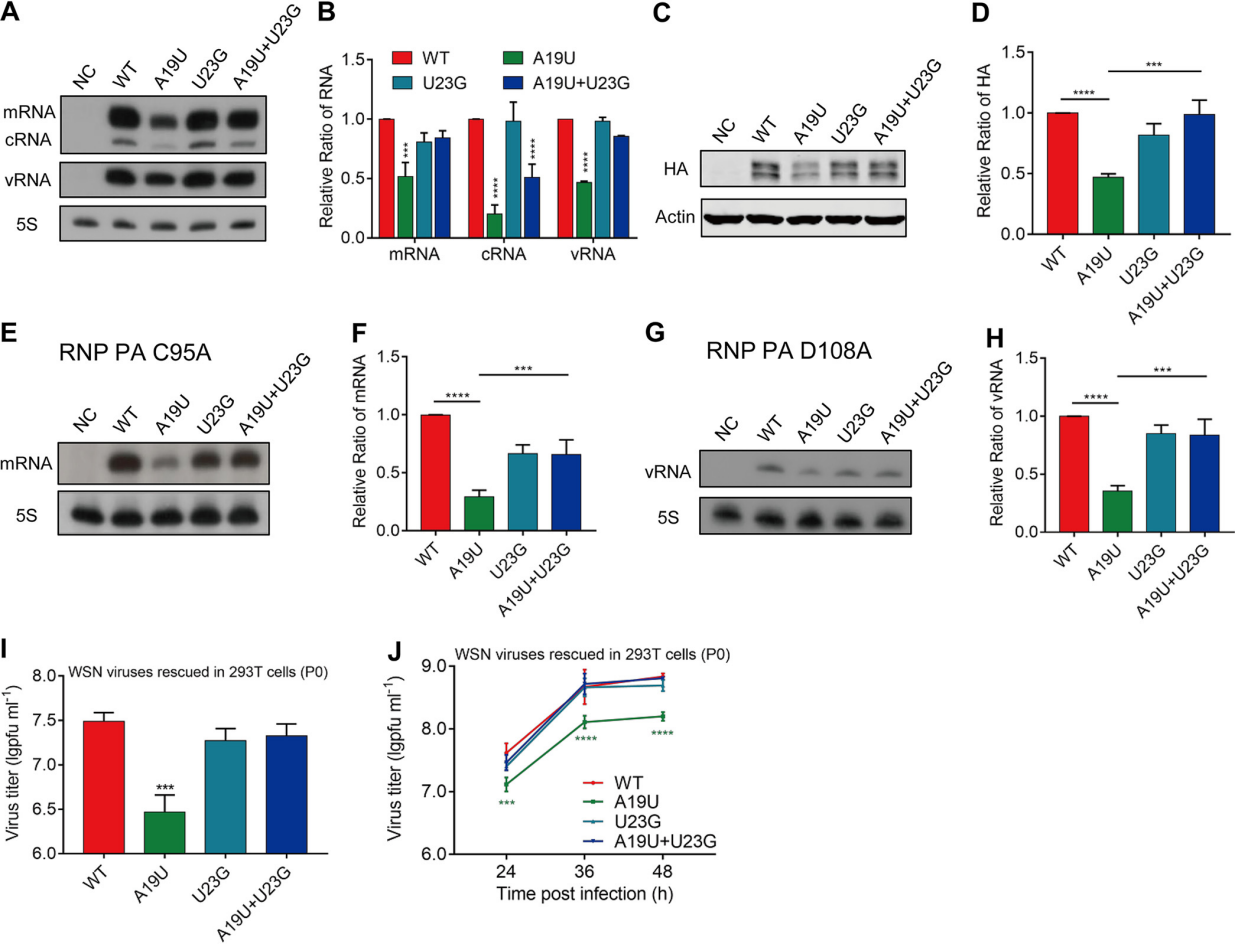
A reverted U23G mutation restores the effects of A19U during virus propagation. (A) Effects of U23G mutation on viral RNA synthesis. 293T cells were transfected with four protein expression plasmids (pcDNA-PA, -PB1, -PB2, and -NP), together with a wild-type or mutant viral RNA expression plasmid (pPOLI-HA). A mutant polymerase containing active-site mutations (PB1a: D445A/446A) was used as a negative control (NC). The levels of mRNA, cRNA, and vRNA were detected by a primer extension assay at 24 h posttransfection. (B) Statistical analysis of the viral RNAs in A. The values of viral RNAs were standardized to the 5S rRNA level and then normalized to the levels of viral RNAs in the wild type. (C) Effect of U23G mutation on viral protein expression. 293T cells were transfected as indicated for A. The levels of the HA protein were analyzed by Western blotting at 24 h posttransfection. β-Actin was detected as a loading control. (D) Quantification of the HA protein levels obtained from the experiments shown in C by densitometry analysis. (E) Effect of the point mutation A19U on viral RNA transcription. 293T cells were transfected with four protein expression plasmids (pcDNA-PA-C95A, -PB1, -PB2, and -NP), together with a wild-type or mutant viral RNA expression plasmid (pPOLI-HA). A mutant polymerase containing active site mutations (PB1a: D445A/446A) was used as negative control (NC). The level of mRNA was detected by a primer extension assay at 24 h posttransfection. (F) Statistical analysis of the viral RNA in E. The values of viral RNA were standardized to the 5S rRNA level and then normalized to the levels of viral RNAs in the wild type. (G) Effect of the point mutation A19U on viral RNA replication. 293T cells were transfected with four protein expression plasmids (pcDNA-PA-D108A, -PB1, -PB2, and -NP), together with a wild-type or mutant viral RNA expression plasmid (pPOLI-HA). A mutant polymerase containing active site mutations (PB1a: D445A/446A) was used as a negative control (NC). The level of vRNA was detected by a primer extension assay at 24 h posttransfection. (H) Statistical analysis of the viral RNA in G. The values of viral RNA were standardized to the 5S rRNA level and then normalized to the levels of viral RNAs in the wild type. (I) Titers of wild-type and mutant WSN rescued viruses (P0). Wild-type or mutant viruses were rescued in HEK-293T cells by cotransfecting eight pHW2000-PB2, -PB1, -PA, -HA (wild type or mutant), -NP, -NA, -M, and -NS plasmids. The supernatants were harvested at 48 h posttransfection, and the titers were determined by plaque assay. The graph shows the mean titers of wild-type and mutant viruses. (J) Replication kinetics of wild-type and mutant viruses in MDCK cells. MDCK cells were infected with P0 generation wild-type or mutant viruses at an MOI of 0.001, and the supernatants were harvested at indicated time points. The virus titers were determined by plaque assay. All data represent the mean ± SD; *n* = 3 biological replicates. ***, *P* < 0.001; ****, *P* < 0.0001.

In order to assess the effect of the point mutation U23G, we constructed the U23G and A19U+U23G mutations in the pPOLI-HA plasmid and examined the effects of these mutants on viral RNA synthesis and protein production in the RNP reconstitution system as described earlier. In general, the results were consistent with the results observed above; the point mutation A19U significantly attenuated the level of all three species of viral RNAs, while the A19U+U23G mutation significantly increased the HA vRNA and mRNA levels compared with A19U mutation (Fig. 5A and B). Of note, we found that although the A19U+U23G mutation restored both HA mRNA and vRNA levels to the wild-type level, the cRNA level was still lower than that of the wild type (Fig. 5A). We speculated that the reduced cRNA level is sufficient to produce vRNA and the mutations have a specific impact on vRNA→cRNA synthesis. In terms of protein production, the A19U+U23G mutation significantly increased the level of HA protein compared with the A19U mutation which correlates well with the mRNAs of the mutants (Fig. 5C and D). In contrast, the U23G mutation alone did not have any effect on HA RNAs or protein levels (Fig. 5A to D).

Since the A19U mutation was introduced in the 3′ H1-ssNCR of vRNA, it would be used as the template to conduct both viral RNA transcription (vRNA→mRNA) and replication (vRNA↔cRNA); next, we wanted to explore the effects of the A19U mutation on viral RNA transcription and replication, respectively. We first investigated the effect of the A19U mutation on transcription through an RNP reconstitution system which contains a replication-deficient polymerase (PA-C95A) (32) to avoid the generation of cRNA and vRNA. We found that the A19U mutation resulted in a significant decrease in mRNA synthesis, and the U23G mutation rescued the level of mRNA (Fig. 5E and F). Next, we used an RNP reconstitution system, which contains a transcription-deficient polymerase (PA-D108A) (33) to avoid the generation of mRNA, to examine the effect of the A19U mutation on replication. The results showed that the A19U mutation significantly reduced the level of vRNA, and the U23G mutation rescued the level of vRNA (Fig. 5G and H). Besides, given the effects of the three mutant templates (A19U, U23G, and A19U+U23G) on cRNA levels, we could further induce that, during viral RNA replication, A19U may have a major defect in vRNA→cRNA synthesis.

To further assess the effect of the U23G mutation on WSN virus growth efficiency, we generated U23G and A19U+U23G recombinant viruses using the reverse genetics and examined the titers of the rescued viruses (P0) by plaque assay. Unlike the significant attenuation (1 log reduction) observed for the A19U mutant virus, the titer of the A19U+U23G mutant virus was similar to that of the wild-type virus (Fig. 5I). In order to prevent the emergence of the U23G mutation in the A19U mutant virus and to examine the effects of the actual mutation on virus growth, we detected the growth kinetics of the P0 generation wild-type and mutant viruses. The results showed that the titer of the A19U mutant virus decreased about 0.7-log compared with that of the wild-type virus, while there was no significant difference between wild-type and A19U+U23G mutant viruses (Fig. 5J). Together, these results show that the A19U mutation has affected both viral RNA transcription and replication, and the revertant U23G in the H1-ssNCR neutralizes the decrease of the HA RNA levels and virus titers caused by the point mutation A19U.

## DISCUSSION

The segment-specific and subtype-specific NCRs of influenza A virus are highly conserved within each segment but vary significantly in length and sequences between different segments. However, the biological significance of these variations and the sequence requirements remain poorly understood. In this study, we investigated the sequence requirements of the 3′ and 5′ H1-ssNCRs in the context of H1N1 WSN virus. By performing the self-designed vRNSA, it provides, for the first time, a visualized dynamic overview directly reflecting the hierarchical sequence requirements within and between the 3′ and 5′ H1-ssNCRs. The results showed that, between the 3′ and 5′ H1-ssNCRs, the sequence requirements of the 3′ H1-ssNCR in general was more stringent than that of the 5′ H1-ssNCR; within the 3′ and 5′ H1-ssNCRs, sequence stringency is declining with the locations of the nucleotides away from the promoter. Moreover, the stabilized sequences presented a common feature of high A/U ratios. More importantly, we reported that the identities of nucleotides close to the 3′ vRNA promoter cooperatively play a role in regulating viral RNA transcription and replication.

It is widely accepted that the key packaging signal of each segment is composed by ssNCRs and its adjacent terminal coding sequences at both ends. In the studies of packaging signal identifications for each segment of WSN virus through serial truncation of the terminal coding sequences, the authors mentioned that the 3′-terminal coding region is more important than the 5′-terminal coding region toward the efficient packaging of PB2, HA, NA, and NS segments (24–27). Consistent with these findings, we previously replaced the wild-type H1-ssNCRs at the 3′ or 5′ ends with the corresponding ssNCRs of the other HA subtypes (H2- to H7- and H9-ssNCRs) individually and found that the 3′ H1-ssNCR plays a more critical role than the 5′ H1-ssNCR on HA vRNA virion incorporation (13). In this report, our sequence logos produced by the vRNSA further show that the speed of the sequence stabilization in the 3′ H1-ssNCR is generally faster than that of the 5′ H1-ssNCR. Together, these results strongly indicate that the 3′ H1-ssNCR plays a more important role than the 5′ H1-ssNCR for efficient virus replication.

In addition to the disparity on stabilization speed, the actual sequence stringency displayed in the vRNSA logos is also significantly different between the 3′ and 5′ H1-ssNCRs. The nucleotides in 3'G1 showed the exact wild-type sequence in the both replicates at P6 and the nucleotides in 3'G2 and 3'G3 showed constant but non-wild-type sequences. In contrast, the nucleotides in 5'G1 showed one wild-type and one non-wild-type constant sequence and the nucleotides in 5'G2 and 5'G3 were still in disordered status at P6. These results clearly demonstrate that the sequence requirements in the 3′ H1-ssNCR are, in general, more stringent than that those in the 5′ H1-ssNCR; especially, the promoter-proximal nucleotides at both ends are of the greatest importance for the virus to survive. Our in-depth *in vivo* functional analyses demonstrate that the nucleotides adjacent to the promoter region in the 3′ H1-ssNCR cooperatively regulate the HA vRNA synthesis, further highlighting the importance of 3′ promoter-proximal nucleotides in regulating virus replication efficiency. Moreover, we previously studied the functions of the 3′ H1-ssNCR with a series of truncation mutants, and we found that the truncation with these six nucleotides or less (3′ 15 to 20) left could work with its adjacent coding nucleotide toward regulating HA vRNA template preference in a multisegment environment (29). Of note, in our vRNSA experiment, we performed NGS for either the 3′ NCR or 5′ NCR. We could not exclude the possibilities that compensatory mutations in the entire viral genome could occur, which, however, is beyond the scope of this study. Moreover, we showed evidence from both the vRNSA experiment and the A19U mutant virus that the identity of a specific nucleotide (A19) in the 3′ promoter-proximal region plays an important role during viral RNA synthesis. Together, we propose that the nucleotides adjacent to the 3′ promoter region play an essential role in regulating viral RNA synthesis. Nevertheless, further investigation is needed to clarify the detailed mechanisms by deep sequencing and function-related high-resolution RNA structure studies.

Interestingly, our vRNSA logos showed that all stabilized sequences in the 3′ and 5′ H1-ssNCRs at P6 have a common feature of high A/U ratios (>66%) (Table 1). We then checked the sequences of all segment-specific and subtype-specific NCRs and found that all ssNCRs contain A/U nucleotides in a range of 60% to 100%. However, the underlying mechanism(s) remain unclear. Dadonaite et al. (34) investigated the global high-resolution structure of the influenza A virus genome and found that vRNA in the context of vRNP is capable of accommodating secondary RNA structures through extensive base pairing. We speculate that the high occupancy of A and U bases may increase the free energy of the ssNCR structures and thus facilitate the polymerase to unravel the structures to initiate viral RNA transcription and replication.

In summary, by using the self-designed vRNSA, we generate, for the first time, the visualized dynamic sequence selection logos for the 3′ and 5′ H1-ssNCRs during virus rescue and serial passage. It clearly differentiates the importance of the 3′ H1-ssNCR versus the 5′ H1-ssNCR and also demonstrates hierarchical sequence requirements of the nucleotides within the 3′ and 5′ H1-ssNCRs. Our functional assays further pinpoint the essentiality of the promoter-proximal nucleotides in regulating influenza virus replication. These data will not only broaden our knowledge in understanding the nonconserved noncoding region of influenza A virus but also may provide basic information to design the antisense oligonucleotide (ASO) in targeting key viral RNA sequences.

## MATERIALS AND METHODS

### Cells and viruses.

Madin-Darby canine kidney (MDCK), human lung carcinoma cell line A549, and human embryonic kidney 293T (HEK-293T) cells were purchased from the American Type Culture Collection (ATCC) (Manassas, VA) and maintained in Dulbecco’s modified Eagle’s medium (DMEM) (Gibco) supplemented with 10% fetal bovine serum (FBS) (Gibco), 100 U/mL penicillin, and 100 μg/mL streptomycin at 37°C and 5% CO_2_. Influenza A/WSN/33 viruses were kindly provided by Ervin Fodor (University of Oxford, UK).

### Plasmids and antibodies.

The eight plasmids of the influenza virus A/WSN/33 (H1N1) reverse genetic system (pHW2000-PB2, pHW2000-PB1, pHW2000-PA, pHW2000-HA, pHW2000-NP, pHW-2000NA, pHW2000-M, and pHW2000-NS) were described previously (35). The vRNP reconstitution system of influenza virus A/WSN/33/(H1N1) (pcDNA-PB2, pcDNA-PB1, pcDNA-PB1a, pcDNA-PA, pcDNA-NP, and pPOLI-HA) was kindly provided by Ervin Fodor (Oxford University, Oxford, UK) (36). Different H1-ssNCR mutant pHW2000-HA plasmids were constructed by PCR with specific primers using site-directed PCR mutagenesis.

### Reverse genetics.

Virus rescue was conducted by the previously described protocol for the eight-plasmid rescue system (37). Briefly, approximately 10^6^ 293T and MDCK cells (at a ratio of 2:1) in a 6-well plate were transfected with 0.5 μg of each plasmid of the influenza virus A/WSN/33 (H1N1) reverse genetic system (pHW2000-PB2, -PB1, -PA, -HA, -NP, -NA, -M, and -NS) using Lipofectamine 2000 and Opti-MEM according to the manufacturer’s instructions. At 24 h posttransfection, the medium was replaced with DMEM containing 0.5% FBS and 0.5 μg/mL tosylsulfonyl phenylalanyl chloromethyl ketone (TPCK)-treated trypsin (Sigma). The virus supernatant was harvested 48 h after the medium was changed, and the viruses were plaque purified. The purified viruses were passaged for two generations in MDCK cells and subjected to virus genome sequencing. The virus titers were then determined by a standard plaque assay.

### Virus random nucleotide selection assay.

The HA 3′ and 5′ ssNCRs of the WSN virus were divided into three groups. Each group of the 3′ H1-ssNCR contained six random nucleotides (3'G1 [15 to 20], 3'G2 [21 to 26], and 3'G3 [27 to 32]), except that the 3'G3 group contains an unchanged -3U nucleotide. Each group of the 5′ H1-ssNCR contained eight random nucleotides (5'G1 [22' to 29'], 5'G2 [30' to 37'], and 5'G3 [38' to 45']). Nucleotides in each group were randomized by performing PCR with primers containing degenerate nucleotides (3’G1 forward primer, 5′-GCGCGTCTCCGGGAGCAAAAGCAGGGGNNNNNNAAAACAACCAAAATGAAGGCAAAACTACTGGTCCTGTTATATGCATTTG-3′; 3’G2 forward primer, 5′-GCGCGTCTCCGGGAGCAAAAGCAGGGGAAAATANNNNNNACCAAAATGAAGGCAAAACTACTGGTCCTGTTATATGCATTTG-3′; 3’G3 forward primer, 5′-GCGCGTCTCCGGGAGCAAAAGCAGGGGAAAATAAAAACANNNNNNATGAAGGCAAAACTACTGGTCCTGTTATATGCATTTG-3′; 3’G reverse primer, 5′-GCGCGTCTCCGGGAGCAAAAGCAGGGGNNNNNNAAAACAACCAAAATGAAGGCAAAACTACTGGTCCTGTTATATGCATTTG-3′; 5’G forward primer, 5′-GCGCGTCTCCGGGAGCAAAAGCAGGGGAAAATAAAAACAACCAAAATGAAGGCAAAACTACTGG-3′; 5’G1 reverse primer, 5′-GCGCGTCTCCTATAGTAGAAACAAGGGTGTTTTTNNNNNNNNTTCTGAAATCCTAATCTCAGATGCATATTCTGCACTGC-3′; 5’G2 reverse primer, 5′-GCGCGTCTCCTATAGTAGAAACAAGGGTGTTTTTCCTTATATNNNNNNNTCCTAATCTCAGATGCATATTCTGCACTGC-3′; 5’G3 reverse primer, 5′-GCGCGTCTCCTATAGTAGAAACAAGGGTGTTTTTCCTTATATTTCTGAAANNNNNNNNTCAGATGCATATTCTGCACTG-3′). The PCR products were ligated into an empty pHW2000 plasmid by BsmBI sites and then propagated in Escherichia coli strain DH5α, which resulted in at least 4^6^ (each 3′ ssNCR group) or 4^8^ (each 5′ ssNCR group) independent clones per library. We varied six or eight nucleotides for each library given the transformation efficiency in plasmid construction and the transfection efficiency in virus rescue. Then we rescued mixed viruses of the six libraries by the reverse genetics. A total of 10^7^ 293T cells in 10-cm dishes was transfected with 1 μL (1 μg/μL) of each plasmid of the influenza virus A/WSN/33 (H1N1) reverse genetics system (pHW2000-PB2, -PB1, -PA, -HA (one of the six libraries), -NA, -M, and -NS). At 48 h posttransfection, the virus-containing supernatants were harvested and the virus titers were determined by plaque assay. The mixed viruses of each library were propagated for six generations incubated with 10^7^ MDCK cells in T175 flasks at a multiplicity of infection (MOI) of 0.1 for 1 h at room temperature because 10^6^ virus particles far exceed the total number of likelihood in each library. MDCK cells were then washed with phosphate-buffered saline (PBS) twice and overlaid with DMEM containing 0.5% FBS and 0.5 μg/mL TPCK-treated trypsin. When the cytopathic effect (CPE) reached about 50% of the total cells, the supernatants were harvested.

To identify the HA ssNCR sequences required for efficient virus replication selected by this method, viral supernatants of passage 2, 4, and 6 were clarified by centrifugation at 3,000 rpm for 20 min. The supernatant was further centrifuged at 10,000 rpm for 30 min at 4°C. Then, the clarified supernatant was collected and loaded onto a 30% sucrose cushion and further centrifuged at 25,000 rpm for 2.5 h at 4°C. The RNA of the virus pellet was extracted using TRIzol reagent (Invitrogen) and reverse transcribed according to the following method of virus genome sequencing. The reverse transcription (RT) products were then amplified together by PCR with a high-fidelity enzyme using HA 3′ H1-ssNCR-specific primers with different barcode sequences. To avoid excessive cycles of PCR that may affect the actual proportion of bases at different positions of the 3′ and 5′ H1-ssNCRs, we performed quantitative PCR (qPCR) at first with RT products that were diluted 5 times as the template to choose a suitable number of cycles for PCR and make sure that the PCR products were in exponential phase. After performing DNA gel electrophoresis and confirming the locations of lines, we recycled the PCR products and subjected them to deep sequencing. The ratios of different nucleotide identities at respective sites of HA ssNCRs were calculated and analyzed. The ssNCR sequence logos for each library showing the nucleotide frequency were constructed by the online Web server WebLogo (http://weblogo.berkeley.edu/logo.cgi) (38).

### Virus genome sequencing.

The total RNA of each virus stock was extracted with TRI LS reagent (Sigma-Aldrich) and reverse transcribed using the SuperScript III first-strand synthesis system (Invitrogen) with a universal influenza A virus reverse transcription (RT) primer: (a mixture of 5′-GTTCAGACGTGTGCTCTTCCGATCTAGCGAAAGCAGG-3′ and 5′-GTTCAGACGTGTGCTCTTCCGATCTAGCAAAAGCAGG-3′; the natural nucleotide variation in the RT primers is underlined). The RT products were then amplified by PCR using HA NCR-specific primers (3′-end forward primer, 5′-GTGCTCTTCCGATCTAGCAAAAGCAGG-3′; 3′-end reverse primer, 5′-GATGTTACATTTCCCCAATTGTAGTGGGGC-3′; 5′-end forward primer, 5′-CTACCACAAGTGTGACAATG-3′; 5′-end reverse primer, 5′-ATCGCGTCTCCGGGAAGTAGAAACAAGG-3′). Finally, the PCR products were sequenced.

### Growth curve analysis.

MDCK or A549 cells were infected with wild-type or mutant viruses at a multiplicity of infection (MOI) of 0.001. At time points 24, 36, 48, 60, and 72 h postinfection (p.i.), the supernatants were collected. The virus titers were determined by plaque assay in MDCK cells. The growth curves shown are the average results of three independent experiments.

### Western blot and primer extension analysis.

Approximately 10^6^ HEK-293T cells were transfected with 0.5 μg each of four protein expression plasmids (pcDNA-PA, -PB1, -PB2, and -NP) and pPOLI-HA (wild type or mutant) using Lipofectamine 2000 and Opti-MEM according to the manufacturer’s instructions. Cells were harvested 24 h posttransfection. For Western blot analysis, the cells were lysed with Cytobuster lysis buffer (Novagen), followed by Western blotting with a monoclonal anti-HA antibody, with anti-β-actin antibody as an internal control. Protein expression levels were visualized with an Odyssey infrared imaging system (Li-Cor Biosciences, USA). The relative protein expression level was analyzed using the integrated software of the Odyssey system. For primer extension analysis, total RNA was extracted using TRIzol reagent (Invitrogen). The levels of mRNA, cRNA, and vRNA of the HA template were analyzed by primer extension with two ^32^P-labeled HA-specific primers, as follows: 5′-TACTCAACTGTCGCCAGTTC-3′ was used to detect HA vRNA and 5′-GTCACTGCCACATTCTTCTC-3′ was used to detect HA mRNA and cRNA. The primer used for detecting internal control 5S rRNA was 5′-TCCCAGGCGGTCTCCCATCC-3′. Primer extension products were analyzed by 6% denaturing PAGE with 7 M urea in Tris-borate-EDTA (TBE) buffer and visualized by phosphor-imaging on an FLA-5000 scanner (Fuji). ImageJ was used to analyze the ^32^P-derived signal (39).

### Statistical analysis.

GraphPad Prism, version 7, software was used for statistical analysis. Two-way analysis of variance (ANOVA) with Dunnett correction was used for two-variable comparisons, while one-way ANOVA with Dunnett correction was used for one-variable comparisons. *P* values of <0.05 were considered to be significant.
